# Wnt/beta-catenin signaling in embryonic stem cell converted tumor cells

**DOI:** 10.1186/1479-5876-10-196

**Published:** 2012-09-20

**Authors:** Xinrong Peng, Tao Liu, Ying Wang, Qiaoling Yan, Huajun Jin, Linfang Li, Qijun Qian, Mengchao Wu

**Affiliations:** 1 Laboratory of Viral and Gene Therapy, Eastern Hepatobiliary Surgical Hospital, The Second Military Medical University, Shanghai, China; 2 Xinyuan Institute of Medicine and Biotechnology, College of Life Science, Zhejiang Sci-Tech University, Hangzhou, Zhejiang, China

**Keywords:** Embryonic stem cell, Malignancy, Wnt/β-catenin signaling

## Abstract

**Background:**

Embryonic stem cells (ESCs) are pluripotent stem cells and can form tumors containing cells from all three germ layers. Similarities between pluripotent stem cells and malignant tumor cells have been identified. The purpose of this study was to obtain ESCs-converted tumor cell lines and to investigate the mechanism of malignancy in pluripotent stem cells.

**Methods:**

Mouse ESCs were subcutaneously injected into nude mice to obtain tumors from which a tumor-like cell line (ECCs1) was established by culturing the cells in chemical-defined N2B27 medium supplied with two small molecular inhibitors CHIR99021 and PD0325901 (2i). The ECCs1 were then subcutaneously injected into nude mice again to obtain tumors from which another tumor-like cells line (ECCs2) was established in the same 2i medium. The malignant degree of ESCs, ECCs1 and ECCs2 was compared and the underlying mechanism involved in the malignancy development of ESCs was examined.

**Results:**

The three ESCs, ECCs1 and ECCs2 cell lines were cultured in the same 2i condition and showed some likeness such as Oct4-expression and long-term expansion ability. However, the morphology and the tumor-formation ability of the cell lines were different. We identified that ECCs1 and ECCs2 gradually acquired malignancy. Moreover, Wnt signaling-related genes such as CD133 and β-catenin expression were up-regulated and Frizzled related protein (FRP) was down-regulated during the tumor development of ESCs.

**Conclusions:**

The two tumor-like cell lines ECCs1 and ECCs2 stand for early malignant development stage of ESCs and the ECCs2 was more malignant than the ECCs1. Moreover, we identified that Wnt/β-catenin signaling played an important role in the malignancy process of ESCs.

## Background

Embryonic stem cells (ESCs) are pluripotent stem cell which can develop into normal individuals after being injected into blastocysts [1-3]. ESCs can form tumors after being injected into nude mice [4], which is a security barrier for cell replacement therapy of ESCs. Recently, several groups reported that significant signatures related to ESCs were also identified in many human cancers and in mouse cancer models [5-9], indicating that tumorigenesis might start at an early ‘embryonic’ state. Classical views of tumorigenesis have invoked tissue dedifferentiation in the oncogenic process, whereas modern views assume that tumor initiating cells might arise from stem cells or from the dedifferentiation of somatic cells. Somatic cells have been reprogrammed to induced pluripotent stem cells (iPSCs) by transduction of defined transcription factors such as Oct4 [10-13] and the generation of mammalian offspring after somatic cell nuclear transfer (SCNT) have been obtained [14,15]. These results showed that somatic cell can be reprogrammed into ESCs-like pluripotent stem cells and then convert into the tumor initiating cells. Thus, establishment of ESCs-converted tumor cell lines will provide excellent models for researching the mechanism of tumorigenesis and new approaches for cancer therapy. In this study, ESCs was subcutaneously injected into nude mice to form tumors, from which a tumor-like cell line (ECCs1) was derived. ECCs1 were then subcutaneously injected into nude mice to form tumors again, from which another tumor-like cell line ECCs2 was established. Our results indicated that ECCs1 showed a tendency of malignant and ECCs2 was more malignant than ECCs1. Furthermore, ECCs1 and ECCs2 could maintain Oct4-expression. Recent researches have already examined the overlapping features that are shared by ESCs and tumor cells [8,16,17], indicated that expression of Oct4 is potentially correlated with malignancy and may impact on some aspects of tumor behavior. Moreover, we also found that a cancer stem cell marker [18] CD133 expressed in ECCs2 which is regarded as a Wnt/β-catenin target gene. We then validated the expression of several key target genes in Wnt/β-catenin signaling pathway. It showed that β-catenin up-regulated during the malignance of ESCs and expression of Frizzled related protein (FRP) down-regulated with the progress of malignancy. FRP interact with Wnt proteins, antagonizing Wnt/β-catenin signaling [19]. Wnt/β-catenin signaling improved Oct4-expression and self-renewal ability of human and mouse ESCs [20]. Furthermore, Wnt/β-catenin signaling play a key role in differentiation and maintenance of ESCs *in vitro*[21], and it also play a central function in transforming normal stem cells into cancer stem cells [22-24], all of which verified that Wnt/β-catenin signaling can be utilized both in the acquisition of pluripotency and in tumorigenesis.

Although some similarities between the pluripotent stem cells and tumor cells have been identified, ESCs-converted tumors cell lines need to be established to study the mechanism involved in the earliest tumorigenesis, moreover, further researches need to be performed to solve difficult problems in this field including the currently culture system for ESCs and tumor cells are different. In this study, we maintained mouse ESCs in chemical defined N2B27 medium supplied with two small molecular inhibitors CHIR99021 and PD0325901(2i) as previously described [25]. The ECCs1 and ECCs2 were also maintained in the same chemical-defined 2i medium. Moreover, the two cell lines maintained long-term self-renewal ability in 2i and spontaneously obtained malignancy without transferring viral or transcription factors. We believe that establishment of these cell lines may provide concise models for researching the mechanism of carcinogenesis in ESCs.

## Methods

### Maintenance of ESCs

The ESCs were maintained in N2B27 medium supplied with two inhibitor (2i): CHIR99021 (STEMGENT) and PD0325901 (STEMGENT) as previously described [25]. Other culture medium and growth factors were purchased from Invitrogen. The ESCs were detached using 0.05% trypsin every three days, with a split ratio of 1 to 10. N2B27 is a 1:1 mixture of Dulbecco’s minimal essential medium (DMEM)/F12 and Neurobasal medium supplied with 1% Glutamax, 1 × N2 and 1 × B27. 2i is N2B27 medium supplied with 3 μm/ml CHIR99021 and 0.4 μm/ml PD0325901. The culture medium was changed by half every day. Cells were maintained at 37°C, 5% CO2 and 100% humidity.

### Tumors induction and establishment of ECCs1

Nude mice were purchased from Shanghai Experimental Animal Center, Chinese Academy of Sciences. All animal experiments were carried out in adherence with the National Institutes of Health Guidelines on the Use of Laboratory Animals and approved by the Second Military Medical University Committee on Animal Care (EC11-055). The ESCs were dissociated into single-cell and suspended in phosphate-buffered saline (PBS), each nude mouse was subcutaneously injected 5 × 10^6^ cells to produce tumor. When the diameter of tumors was up to 1.5 cm, the mice were sacrificed. The tumors were removed from the sacrificed nude mice and washed each three times in PBS and then cut into 1cubic millimeter pieces. After digesting by collagenase IV for 30 min at 37°C, the cell suspension was filtered through 200-mesh sieve and centrifuged for 3 min at 100 g. Then the cells were suspended in 2i medium and plated onto 96-well plate in single cell, several days later, the single cell-formed clone was picked up and propagated. The established cell line was named ECCs1.

### Establishment of ECCs2

Cells from ECCs1 were dissociated and suspended in PBS, each nude mouse was subcutaneously injected 5 × 10^6^ cells to produce tumor. When the diameter of tumors was up to 1.5 cm, the mice were sacrificed. Derivation of cells from the ECCs1-converted tumors was same with the derivation of ECCs1. The cells were cultured in 2i medium in single cell, several days later, the single cell-formed clone was picked up and propagated. The established cell line was named ECCs2.

### Malignancy analysis of ESCs, ECCs1 and ECCs2-formed tumors

Differentiation ability of ESCs, ECCs1 and ECCs2 was determined by switching these cells from 2i medium to DMEM medium supplied with 10% fetal bovine serum (FBS). For hematoxylin and eosin (H&E) staining, tissues from ESCs, ECCs1 and ECCs2-converted tumors were separately preserved in 4% paraformaldehyde for 24 h, and then the samples from each group were embedded in paraffin, sectioned, and stained with H&E. Karyotype analysis was performed according to previous study [26].

### Immunostaining assay

We fixed the ESCs, ECCs1 and ECCs2 cells in 4% paraformaldehyde for 20 min at 20°C individually, then washed the cells three times × 10 min with PBS and incubated them in blocking buffer (BB)(0.2% Triton X-100, 1% BSA) in PBS for 1 h at 20°C. Then, the cells were separately incubated with a primary antibody, anti-goat Oct4 (Santa Cruz, sc-8629) or anti-mouse CD133 (eBioscience, 14133180) at a dilution of 1:200 for 12 h at 4°C, the cells were then washed three times × 10 min with PBS and incubated with secondary antibody at a dilution of 1:500 for 1 h at 20°C and washed three times × 10 min with PBS. The cells were incubated with 0.5 μg/ml diamino phenyl indole (DAPI) for five min for staining of nuclei for total cell content.

### Western blot analysis

All the cell lysates (5 × 10^6^) were collected and homogenized in radioimmunoprecipitation buffer (Pierce) supplied with protease inhibitor cocktail (Roche) for 30 min at 4°C. Protein concentrations were determined by a Bicinchoninic Acid Protein Assay Kit (Thermo) and the amounts of protein were normalized for quantification. Protein preparations were resolved on a 10% SDS-polyacrylamide gel and then transferred to nitrocellulose membranes (Protran, Schleicher and Schuell). The membranes were rinsed and probed with the following antibodies at a dilution of 1:1000: β-catenin (BD Transduction Laboratories, 610153), FRP1 (Santa Cruz, sc-13939), standard was tubulin (Sigma T9026,). Blots were visualized using enhanced chemiluminescence (Amersham) and quantified with a Kodak gel documentation system. The Western data were analysised and quantified by using GeneSys software (SynGene)

### Cell growth curve

Cell growth curves of ESCs, ECCs1 and ECCs2 in different culture medium (2i or DMEM supplied with 10%FBS) were determined. Cells from each group were separately cultured in 24-well plates at a density of 1,000 cells per well. Every 24 h, three of the wells from each group were harvested using 0.05% trypsin and the number of cells per well was counted with a hematocytometer. This procedure was repeated for 7 days.

### Statistical analysis

Statistical analysis was performed by using SPSS 11.0. Results are presented as means and standard deviation (SD), and a value of P < 0.05 was considered statistically significant. The paired Student *t* test was calculated to compare the values between each group.

## Results and discussion

### ESCs, ECCs1 and ECCs2 maintained Oct4 expression in 2i medium

**Figure 1:**
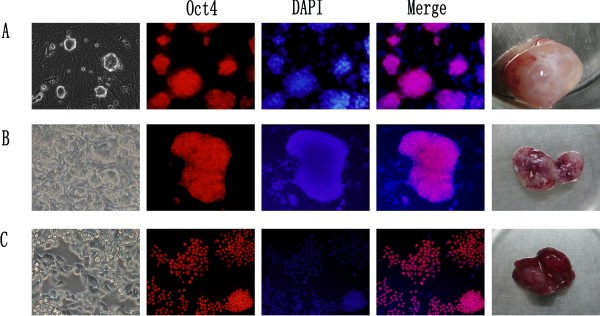
**ESCs, ECCs1 and ECCs2 in 2i medium.** (**A**) ESCs in 2i (left), immunostaining of Oct4 (red), ESCs-converted tumors (right). (**B**) ECCs1 in 2i (left), immunostaining of Oct4 (red), ECCs1-converted tumors (right). (**C**) ECCs2 in 2i (left), immunostaining of Oct4 (red), ECCs2-converted tumors (right). DAPI (blue) staining of nuclei for total cell content.

The mouse ESCs were cultured in chemical-defined 2i medium as previously described [25]. Figure 1A showed that the ESCs grew in clone and maintained Oct4-expression in 2i medium. The ESCs stably maintained their characteristics over numerous passages in 2i medium. After being injected into nude mice, the ESCs formed tumors measuring up to 1.5 cm in diameter within 7–8 weeks (Figure 1A). Then, the mice were sacrificed and tissues obtained from the tumors were dissociated into single cell and cultured in 2i medium. The dissociated cells grew abundantly in 2i medium. We picked up a single cell-formed clone and named the clone ECCs1 (Figure 1B). Although the ECCs1 was cultured in the same culture condition with the ESCs, the ECCs1 displayed cellular disarrangement and the morphology of ECCs1 was gradually different with the ESCs (Figure 1B). The ECCs1 also maintained Oct4-expression in 2i medium (Figure 1B). We already maintained the cells more than 50 passages and the cells still grew abundantly in 2i medium. Then, we injected the ECCs1 into nude mice again. The ECCs1 formed tumors measuring up to 1.5 cm in diameter within 6–7 weeks (Figure 1B). The mice were then sacrificed and tissues from the ECCs1-formed tumors were dissociated into single cell and cultured in 2i medium. Several days later, a single-cell clone was picked up and named as ECCs2 whose morphology was completely different from that of ESCs (Figure 1C). The ECCs2 did not grow in separate clone but showed overlapping growth and cellular disarrangement. As well as ESCs and ECCs1, Figure 1C showed that the ECCs2 still maintained Oct4-expression in 2i medium and long-term self-renewal ability. After being injected into nude mice, the ECCs2 could form tumors measuring up to 1.5 cm in diameter within 4 weeks. Taken together, these results indicated that ECCs1 and ECCs2 were different from ESCs and showed a tendency of malignancy.

It is well known that ESCs could form tumors after being injected into nude mice and the tumors are mainly composed of normal cells and tissues from three germ layers [4]. However, our results indicated that Oct4-possitive cells still existed in the ESCs-formed tumors. Moreover, in our previous study, we also injected a well established Oct4-GFP knock-in mouse ESCs [27] into nude mice to form tumors and several Oct4-positives cell lines were derived from the tumors by using the 2i medium [28]. We purposed that there were several factors that facilitated the derivation of the Oct4-possitive cells. First, in this study, we cut the ESCs-formed tumors into 1cubic millimeter pieces and different samples from the tumors were collected for deriving ECCs1 and ECCs2. Second, most of differentiated cells could not survive in the serum-free 2i medium. Third, the 2i medium includes small molecular inhibitor CHIR99021 and PD0325901. CHIR99021 is a GSK3 inhibitor [29] and previous studies showed that inhibition of GSK3 plays an important role in maintaining Oct4-expression and overall cell survival ability [20,25]. PD0325901 is an inhibitor of mitogen-activated protein kinase signaling which induces differentiation of ESCs [30]. The 2i medium was first applied to derive mouse ESCs [25]. In this study, we applied 2i medium to derive and maintain the two ECCs1 and ECCs2 cell lines. Although we cultured the ESCs, ECCs1 and ECCs2 in the same 2i culture condition, the morphology and the tumor-formation ability of the three cell lines were totally different (Figure 1). In our previous study, we also performed microarray analysis to show that many well-known tumor-associated genes change with the malignancy development of ESCs after sequential transplantation [28]. Taken together, these results indicated that the subcutaneous microenvironment of nude mice could accelerate the malignancy degree of ESCs. We proposed that the two tumor-like ECCs1 and ECCs2 cell lines might stand for the early tumor development stage of ESCs.

### ECCs1 and ECCs2 gradually acquired malignant

**Figure 2:**
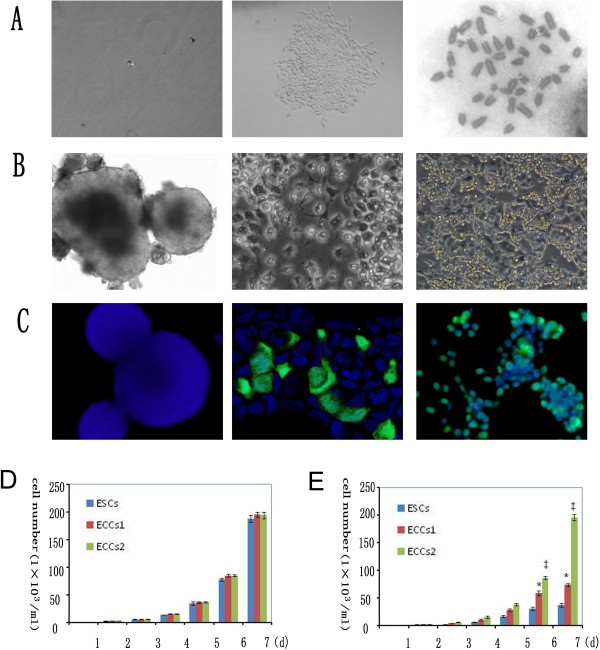
**ESCs, ECCs1 and ECCs2 in DMEM supplied with 10% FBS.** (**A**) tumor-derived cells seeded as single cells (left) Single cell-formed ECCs2 clone (middle), ECCs2 maintained normal karyotype at early passage(right). (**B**) ESCs (left), ECCs1 (middle) and ECCs2 (right) in DMEM supplied with 10% FBS. (**C**) Immunostaining of Oct4 (green) and DAPI (blue) of ESCs (left), ECCs1 (middle) and ECCs2 (right). (**D**) Growth curve of ESCs, ECCs1 and ECCs2 in 2i medium. (**E**) Growth curve of ESCs, ECCs1 and ECCs2 in DMEM supplied with 10% FBS. *,P < 0.05 versus ESCs;‡, P < 0.05 versus ESCs and versus ECCs1. Each point represents a mean of triplicate values for each sample ± SD.

The two tumor-like lines ECCs1 and ECCs2 were established via monoclonal formation (Figure 2A). Even after repeated passage in 2i medium, the karyotype analysis showed that the ECCs2 maintained normal chromosome number (Figure 2A) at early passage (Passage 19).

We then evaluated the likeness and difference among ESCs, ECCs1 (Passage 14) and ECCs2 (Passage 15). The three cell lines shared several same characteristics such as Oct4-expression and self-renewal ability in 2i medium. However, after withdrawal of 2i, the ESCs could not maintain long-term self-renewal ability. The ESCs differentiated into embryonic body in DMEM medium supplied with 10% FBS (Figure 2B) and Oct4-expression of the ESCs quickly disappeared (Figure 2C). The results confirmed that the ESCs maintained a balance of self-renewal and differentiation, after withdrawal of 2i inhibitors, the ESCs quickly converted into normal differentiated cells. We observed that the ECCs1 grew abundantly and differentiated partially in DMEM medium supplied with 10% FBS (Figure 2B), some of which still maintained Oct4-expression for 2–3 passages (Figure 2C). The results indicated that the ECCs1 had a tendency of malignancy. After withdrawal of 2i, the ECCs2 maintained Oct4-expression in DMEM medium supplied with 10% FBS (Figure 2C) even after long-term expansion and repeated passages (Figure 2B) which verified that ECCs2 lost the appropriate regulatory mechanisms to maintain a balance of self-renewal and differentiation.

The cell growth curve assay confirmed that ESCs, ECCs1 and ECCs2 grew abundantly and maintained exponential growth in 2i medium (Figure 2D) with statistically no significant difference among each group. After withdrawal of 2i, the cell growth curve showed that the differentiated ESCs had two phases of growth: an exponential phase with a steady-state exponential growth and a contact-inhibition phase with cell proliferation cease (Figure 2E). The growth curve of ECCs1 also showed the growth rate of ECCs1 was significant (P < 0.05) higher than that of ESCs (Figure 2E). However, the cell growth curve of ECCs2 only showed an exponential growth phase in DMEM supplied with FBS (Figure 2E) and the growth rate of ECCs2 was significant higher (P < 0.05) than that of ESCs and ECCs1 (Figure 2E). Taken together, these results showed that ECCs2 acquired malignancy and lost differentiation ability.

**Figure 3:**
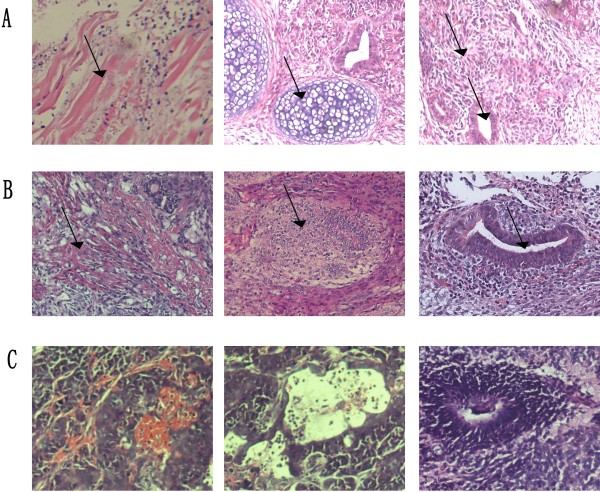
**H&E staining.** (**A**) Muscle (left), bone (middle), neural and gland (right) tissues in ESCs-formed tumors. (**B**) Muscle (left), bone (middle) and gland (right) tissues of ECCs1-formed tumors. (**C**) ECCs2-formed tumors.

We then fixed tissues from the ESCs, ECCs1 and ECCs2-converted tumors to examine the malignancy degree of these cell lines. The H&E staining results showed that the ESCs-formed tumors were mainly composed of normal cells and tissues from the three germ layers (Figure 3A) including muscle, bone, glandular and neural tissues. The ECCs1-formed tumors still included tissues such as muscle, gland and bone, however, the tumors showed a malignant tendency of hyperchromatic nuclei and cellular derangement (Figure 3B). The ECCs2-formed tumors were mainly composed of poorly differentiated adenocarcinoma and carcinomatous nerve tissue (Figure 3C). Moreover, the H&E staining results confirmed that ECCs1 showed a tendency of malignancy and ECCs2 was malignant tumor cells. Therefore, we assumed that ECCs1 was intermediates between the ESCs and ECCs2. These results also indicated that the characteristics of these cell lines were profoundly influenced by microenvironment and the subcutaneous microenvironment of nude mice accelerated the malignancy degree of the ESCs. On the other hand, embryonic microenvironment suppresses the tumorigenic phenotype of aggressive cancer cells proved by several previous works [31,32]. Other studies showed that there is a convergence between malignant tumor cells and embryonic cells in the molecular messengers including members of Notch and Wnt signaling [33,34]. Our results also excluded the possibility that the tumorigenesis of ECCs2 was caused by the loss of chromosomes.

We then evaluated the chromosome number of ECCs2 at late passage (Passage 53). Interestingly, the ECCs2 was established by clone-formation and maintained normal chromosome number at early passage (Figure 2A), however, ECCs2 showed a loss of chromosomes at late passage (Passage 53) and chromosome number of some ECCs2 was around 38 ~ 39 (Additional file 1: Figure S1). We proposed that deletion and amplification of genes and signaling played a role in increasing the rate of chromosome mutations and then the abnormal mitotic mechanisms may result in numerical aberrations in the daughter cells. In this study, we established two ESCs-originated tumor-like cell lines in which ECCs1 showed a tendency of malignancy and ECCs2 was malignant tumor cells. The two cell lines provided models for studying the convergence of tumorigenic and embryonic signaling pathways and then might help to identify new targets for therapeutic intervention.

### Wnt/Î²-Catenin signaling has a relationship with the malignancy of ESCs

**Figure 4:**
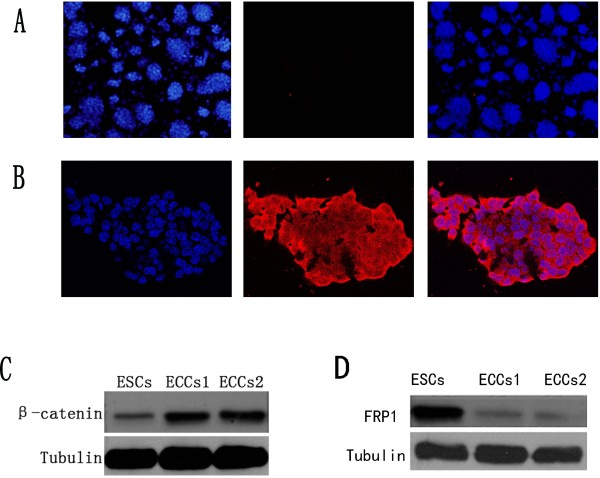
**Wnt/β-catenin signaling in carcinogenesis of ESCs.** (**A**) Immunostaining of DAPI (blue) and CD133 (red) in ESCs. (**B**) DAPI (blue) and CD133 (red) in ECCs2. (**C**) β-catenin-expression in ESCs, ECCs1 and ECCs2. (**D**) FRP1-expression in ESCs, ECCs1 and ECCs2.

To clarify the difference between ESCs and ECCs2, we performed immunostaining to detect the expression of CD133, a common marker for the identification of stem cells from normal and cancerous tissues [35,36]. The immunostaining results showed that CD133 expression in ESCs was negative (Figure 4A), however, most of ECCs2 showed expression of CD133 (Figure 4B), the results also indicated that the ECCs2 was different from the ESCs.

CD133 is regarded as a target gene of Wnt/β-catenin signaling pathway [18]. Previous researches point out that inappropriate activation of the Wnt/β-catenin pathway implicate in transforming normal stem cells into cancer stem cells [22-24,37], however, the role of Wnt/β-catenin signaling in ESCs carcinogenesis remains unclear. In our previous study [28], we also noted that some of Wnt signaling-associated genes changed with the malignancy development of ESCs. We then examined the expression of β-catenin in ESCs, ECCs1 and ECCs2. The western blot results showed that expression of β-catenin was strongly activated in the ECCs1 and ECCs2 compared with ESCs (Figure 4C). The Western data were also quantified by using GeneSys software and the results confirmed that Wnt/β-catenin signaling was activated during malignant development of ESCs (Additional file 2: Figure S2). Furthermore, we also detected another Wnt signaling related protein, FRP1. It is reported that FRP antagonize with Wnt signaling and down-regulation of FRP activate Wnt signaling [19]. The western blot result showed that FRP1 was obviously down-regulated in ECCs1 and ECCs2 (Figure 4D). Expression of FRP1 was also quantified by using GeneSys software and the results confirmed that expression of FRP1 was gradually reduced during the malignant development of ESCs (Additional file 2: Figure S2). According to the results, we assumed that β-catenin might act as a molecular switch in carcinogenesis of ESCs. Possible function of Wnt/β-catenin signaling might include up-regulation of target gene, disruption of linked signaling, deregulation of cell proliferation and disruption of cell adhesion [38]. In summary, our results indicated that down-regulation of FRP1 activated Wnt/β-catenin signaling, up-regulation of β-catenin facilitated the expression of Oct4 in ECCs1 and ECCs2 and then promoted malignancy of these cells. However, it was still unclear which gene regulated the expression of FRP1 during the carcinogenesis of ESCs. In future, we would study and compare more involved genes in Wnt/β-catenin signaling. Also, the iPSCs can form tumors in nude mice [11,12]. It will be useful to establish iPSCs-converted tumor cell lines and our work provides a simple protocol for establishing the pluripotent stem cells-converted tumor cell lines. The chemical-defined culture condition will provide a precise platform for studying the carcinogenesis mechanism of pluripotent stem cells.

## Conclusions

In this study, we established two ESCs-originated tumor-like cell lines ECCs1 and ECCs2, the ECCs1 and ECCs2 could maintain long-term self-renewal ability in 2i medium. Second, our results showed that the ECCs2 was malignant tumor cells and ECCs1 was intermediates between the ESCs and ECCs2, while subcutaneously injection in nude mice accelerated the malignancy of these cells. Third, our work also indicated that Wnt/β-catenin signaling played an important role in the process of malignancy of these cell lines.

## Abbreviations

ESCs: Embryonic stem cells; GFP: Green fluorescent protein; GSK3: Glycogen synthase kinase 3; iPSCs: Induced pluripotent stem cells; PBS: Phosphate-buffered saline; H&E: Hematoxylin and eosin; FRP: Frizzled related protein; DMEM: Dulbecco’s minimal essential medium; DAPI: Diamino phenyl indole; FBS: Fetal bovine serum.

## Competing interests

The authors declare that they have no competing interests.

## Authors’ contributions

XRP carried out cell culture experiment and drafted the manuscript. TL participated in molecular genetics study and carried out animal experiment. YW carried out experimental design. QLY drafted the manuscript. HJJ participated in molecular genetics study. LFL carried out cell culture experiments. QJQ participated in the study design and coordination. All the authors read and approved the final manuscript.

## Supplementary Material

Additional file 1 **Figure S1.**Karyotype analysis of ECCs2 at passage 53. Click here for file

Additional file 2 **Figure S2.**Western data were quantified by using GeneSys software. The value of spot1 ~ 12 stands for the western blot data respectively, Spot 13 stands for the negative control. Click here for file
