# Supplementary figures and images for: Wnt/beta-catenin signaling in embryonic stem cell converted tumor cells
Source: J Transl Med. 2012 Sep 20;10:196. doi: 10.1186/1479-5876-10-196 (PMC3515512; doi:10.1186/1479-5876-10-196)

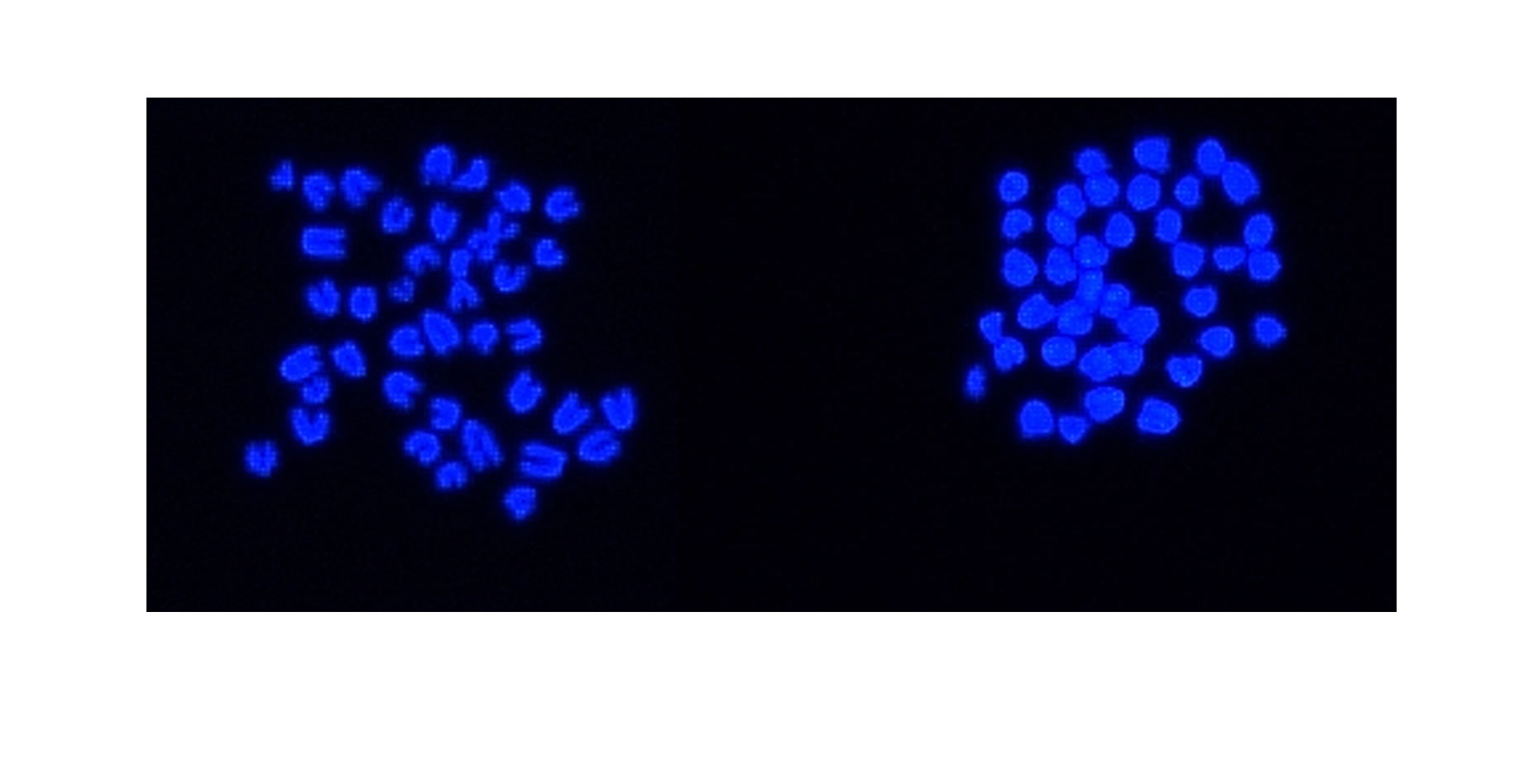

Supplement: Additional file 1 — Figure S1.Karyotype analysis of ECCs2 at passage 53. [file 1479-5876-10-196-S1.jpeg]

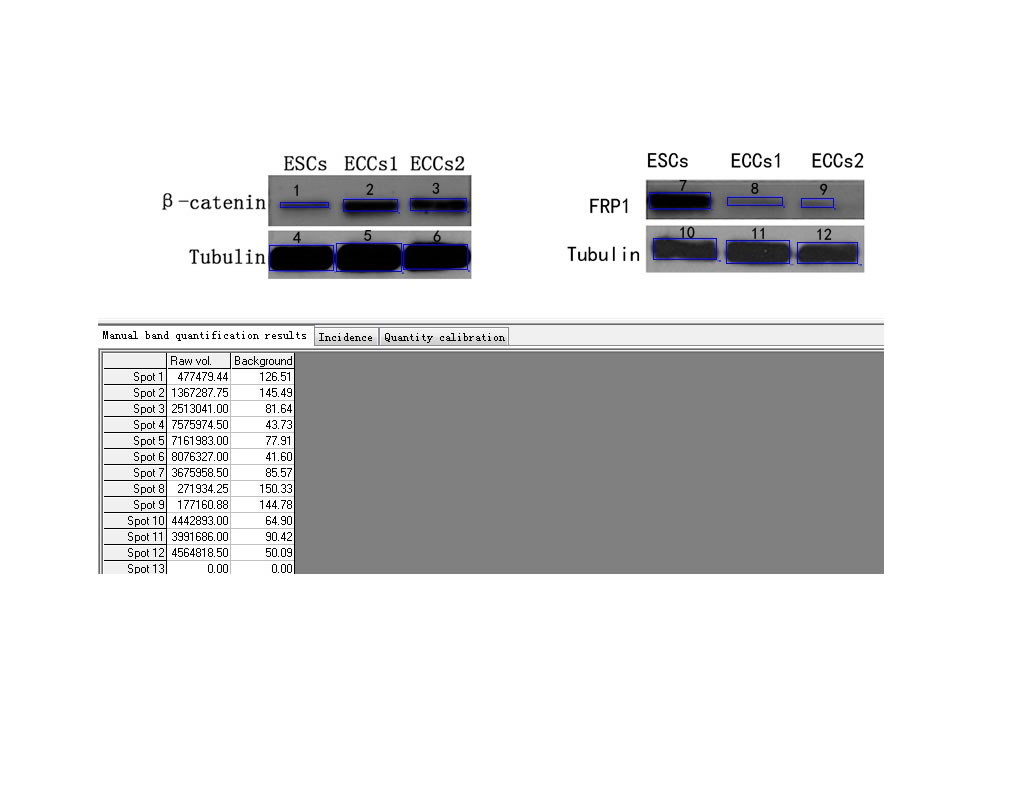

Supplement: Additional file 2 — Figure S2.Western data were quantified by using GeneSys software. The value of spot1 ~ 12 stands for the western blot data respectively, Spot 13 stands for the negative control. [file 1479-5876-10-196-S2.jpeg]
